# The Simple Method of Preparation of Highly Carboxylated Bacterial Cellulose with Ni- and Mg-Ferrite-Based Versatile Magnetic Carrier for Enzyme Immobilization

**DOI:** 10.3390/ijms22168563

**Published:** 2021-08-09

**Authors:** Radosław Drozd, Magdalena Szymańska, Katarzyna Przygrodzka, Jakub Hoppe, Grzegorz Leniec, Urszula Kowalska

**Affiliations:** 1 Department of Microbiology and Biotechnology, Faculty of Biotechnology and Animal Husbandry, West Pomeranian University of Technology in Szczecin, 45 Piastów Avenue, 71-311 Szczecin, Poland; magdalena.szymanska@zut.edu.pl (M.S.); pk36158@zut.edu.pl (K.P.); 2 Faculty of Chemistry, Adam Mickiewicz University, UL. Umultowska 89b, 61-614 Poznań, Poland; kubahoppe@gmail.com; 3 Poznan Science and Technology Park, Adam Mickiewicz University Foundation, Rubież 46 Str., 61-612 Poznan, Poland; 4 Department of Mechanical Engineering and Mechatronics, West Pomeranian University of Technology in Szczecin, 48 Piastów Avenue, 70-311 Szczecin, Poland; grzegorz.leniec@zut.edu.pl; 5 Center of Bioimmobilisation and Innovative Packaging Materials, Faculty of Food Science and Fisheries, West Pomeranian University of Technology in Szczecin, 35 Klemensa Janickiego Str., 71-270 Szczecin, Poland; urszula.kowalska@zut.edu.pl

**Keywords:** bacterial cellulose, magnetic carrier, carboxylation, citric acid, immobilization

## Abstract

The bacterial cellulose (BC) is a versatile biopolymer of microbial origin characterized by high purity and unusual water and material properties. However, the native BC contains a low number of functional groups, which significantly limits its further application. The main goal of its effective modification is to use methods that allow the unusual properties of BC to be retained and the desired functional group to be efficiently introduced. In the present study, the new magnetic carrier based on functionalized citric acid (CA) bacterial cellulose was developed and tested to support critical industrial enzymes such as lipase B from *Candida antarctica* and phospholipase A from *Aspergillus oryzae*. The applied method allowed BC to be effectively modified by citric acid and a sufficient number of carboxylic groups to be introduced, up to 3.6 mmol of COOH per gram of dry mass of the prepared carrier. The DSC and TGA analyses revealed carrier stability at operational temperatures in the range of 20 °C to 100 °C and substantially influenced the amount of the introduced carboxyl groups on carrier properties. Both enzymes’ immobilization significantly improves their thermal stability at 60 °C without a significant thermal and pH optima effect. The analyzed enzymes showed good operational stability with a significant residual activity after ten cycles of repeated uses. The new magnetic carrier based on highly carboxylated bacterial cellulose has a high application capability as matrix for immobilization the various enzymes of industrial interest.

## 1. Introduction

Bacterial cellulose is a unique biopolymer produced among others by the bacteria of the genus *Komagataeibacter xylinus*. Chemically, it is a glucose polymer with the same composition as plant-derived cellulose. However, it has no impurities such as lignins or other types of polysaccharides, and its valorization process for further use is simple and relatively inexpensive [1,2]. This biopolymer has found many applications as an excellent carrier for various active substances, enzymes, or even whole microorganisms [3,4,5,6,7]. However, the native chemical structure of this biopolymer does not ensure a permanent binding of the enzyme to BC. It is mainly based on weak electrostatic, hydrophobic interactions and hydrogen bonds formed by the -OH residues of the primary alcohols and carboxyl groups found in a small amount at the ends of the fibrils formed during the purification of BC. These interactions are susceptible to changes in pH, ionic strength, the presence of specific salts, or temperature changes, and even mixing conditions [8,9,10,11,12]. Properties of the native form of BC, such as porosity, the degree of nanofibrils’ cross-linking, and other material properties, affect the degree of enzyme adsorption and absorption, depending on the culture’s duration and the composition of the culture medium [13,14,15]. To extend the possibility of using BC as a carrier for enzymes, it is subjected to various types of modification. One solution is to add various types of polymers to the culture medium, already at the production stage in cultures, which have functional groups that allow for stronger binding of enzymes to the carrier [16,17,18]. Another solution is composites consisting of purified BC and natural polymers such as chitosan, pectin, alginate, or starch, the addition of which to cellulose modifies the porosity of the material and, above all, facilitates the permanent binding of the enzyme to this type of a carrier [19,20,21]. However, this type of approach, despite its simplicity, requires a further expenditure of materials. In the case of some polymers, there is a problem of their solubility, forcing the selection of appropriate methods to obtain a homogeneous structure of the composite, allowing for the preservation of the unique properties of BC fibrils [22]. Theoretically, the optimal carrier structure should not cause undesirable changes in the operational properties of the immobilized enzyme [23,24]. Despite the possibility of modifying the porosity of composite carriers with BC, there is often a problem disturbing the mass exchange process despite introducing other functional groups and increasing the active surface. The immobilized enzyme shows an increase in the value the K_M_ constant, indicating the problematic contact with both the substrate and the exchange of the resulting product [15,25,26]. The solution to this problem is the effective modification of the BC membrane surface by introducing various types of functional groups, e.g., amine or carboxyl groups as specific anchors that allow a spacer to be added, a linker between the carrier and the enzyme, the task of which is to increase the distance from its surface [27]. So far, the structure of BC, to use it as a carrier for enzymes, has been modified, among others, by applying 1–4 butanediol diglycidyl ether, which allows the introduction of epoxy groups enabling the covalent binding of glucoamylase with the carrier [28]. Another way to modify BC is, for example, its oxidation with the use of periodic acid, in the reaction of which dialdehydes are formed that can be further used as an anchor for polyethyleneimine [29]. An additional advantage of the optimal carrier is the possibility of its easy separation from the reaction environment. A way to simplify the separation of BC-based carriers is to incorporate magnetic field-sensitive materials into their structure [30]. Due to the possibility of modifying their structure, these materials can be characterized by different magnetic susceptibility and properties in the magnetic field. It significantly increases the possibility of using this type of carrier because of the ease of separation with magnetic separators and the possibility of using them in reactors supported by various types of magnetic fields.

The study aimed to prepare new highly carboxylated, magnetic-field-sensitive, bacterial-cellulose-based carriers to immobilize industrially essential enzymes.

## 2. Results and Discussion

### 2.1. The Carriers Properties

Attenuated Total Reflection Fourier Transform Infrared (ATR-FTIR) Spectroscopy Analysis of Carriers’ Properties

**Figure 1:**
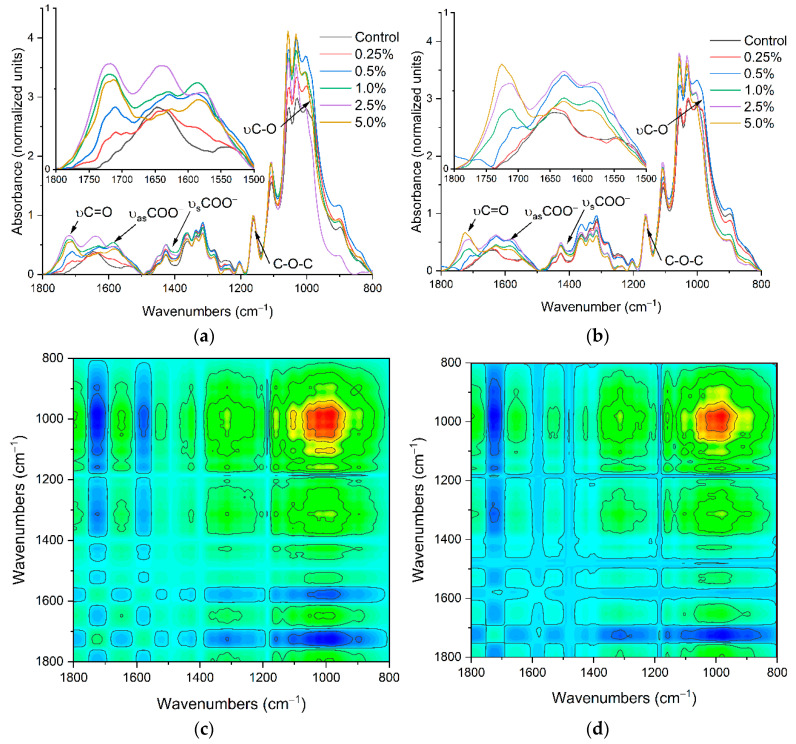

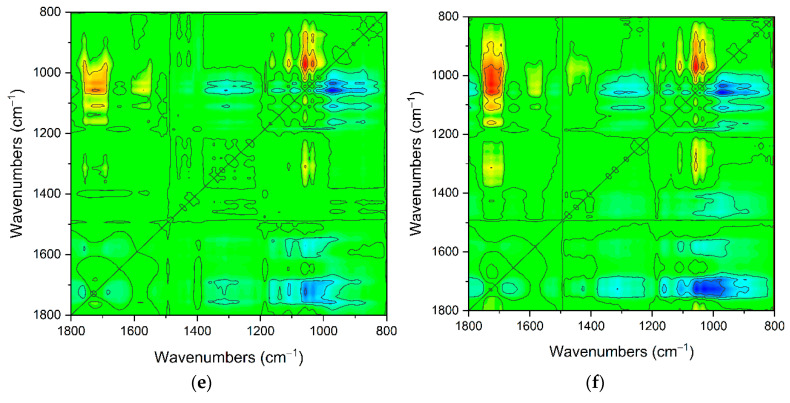
The ATR-FTIR spectra of non-modified bacterial cellulose and its cellulose magnetic composites obtained after modification with the different initial concentrations of CA and types of magnetic particles (**a**) NiFe_2_O_4_, (**b**) MgFe_2_O_4_, and a 2D synchronous and asynchronous spectra for composites with nickel (**c**,**e**) and magnesium (**d**,**f**) ferrites ranging from 1800 cm^−1^ to 800 cm^−1^. Spectra were normalized regarding band area at 1161 cm^−1^ (C–O–C).

The ATR-FTIR spectra confirmed the effectiveness, and CA concentration depended on the modification of BC fibrils (Figure 1a,b). The characteristic bands at 1720 cm^−1^ (C=O, stretching) indicate the occurrence of carbonyl groups from formed ester bonds and COO^−^ [31]. This band appeared from the initial concentration of CA used for composite preparation above 0.5%, and the highest value was presented at the CA amount of 2.5% to 5.0%. The second band appeared only in spectra of modified BC at ≈1580 cm^−1^ assigned to the -O stretch of the deprotonated carboxylic group [32,33,34]. The changes in its intensity were well correlated with changes in the band’s intensity at the range 1760 cm^−1^ to 1710 cm^−1^. Between these bands at approximately 1643 cm^−1^, a visible band was assigned to O–H bending vibration of adsorbed water that overlaps with the 1630 cm^−1^ band specific for COO^−^ carboxylate ion stretching [35,36]. The following bands set from 1450 cm^−1^ to 1320 cm^−1^ are associated with the in-plane OH bending, CH_2_, and CH stretching and bending vibrations. The last bands set at spectra region from 1160 cm^−1^ to 960 cm^−1^ are assigned to C–O–C bridge stretching vibration, asymmetric pyranose ring stretching vibration, and C-O stretching mode of primary and secondary alcohols [37,38,39]. The 2D synchronous spectra of both composites indicate that the most changes occur in two regions from 800 cm^−1^ to 1200 cm^−1^ and 1500 cm^−1^ to 1780 cm^−1^ where significant auto, negative and positive cross-bands appeared (Figure 1c,d). The main significant negative cross-band appeared between 1720 cm^−1^ and ≈980 cm^−1^ assigned to different conformations of primary alcohol at C6-O6H. This site in glucopyranose molecules is most susceptible to modification by CA, which was manifested by reducing this band intensity resulting in ester bond formation [40,41]. Successive visible negative cross-peaks at 1580 cm^−1^, 1400 cm^−1^ vs. ≈980 cm^−1^ also indicated changes in carboxylate amount in a carrier structure. However, variations at region-specific primary alcohol C6-O6H can also indicate the influence of the modification process on BC crystallinity and carrier stability [42].

### 2.2. The Density of the Carboxyl Groups on the Carrier Surface

**Figure 2:**
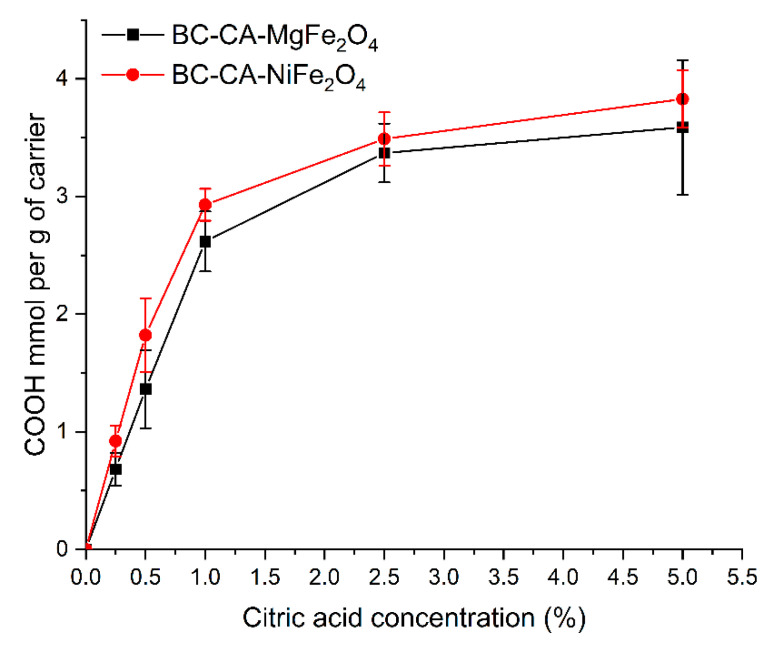
Influence of initial concentration of citric acid on the content of COOH in the structure of bacterial cellulose-based magnetic composites.

The number of carboxyl groups introduced on the carrier surface depended on the initial concentration of CA applied (Figure 2). Both composites had a maximum amount of carboxyl groups at the level of ≈3.6 mmol per g of carriers. The best effectiveness of modification obtained for the initial concentration of CA was 2.5%, above which no further significant increase in carboxyl group content was observed. The type of used magnetic particles did not affect the BC modification process, and both composites exhibited a similar amount of carboxyl groups on their surface. The carboxyl groups density on the carrier surface is critical for its usefulness and efficient enzyme immobilization. Zhu et al. [43] showed that a large amount of carboxyl group on carrier surface such as esterified by citric acid the loofah sponge can reduce the yield of immobilization; however, it is beneficial for enzyme activity. In contrast, Ye et al. [44] reported that the high density of carboxyl groups on carrier surface could negatively influence the activity of immobilized lipase from *Candida rugosa.* The main reason for the reduction in activity could be a result of multisite enzyme immobilization. The enzyme molecule’s multipoint or not directed connection of the activated carrier can lead to changes in its structure or block access to the active site, lower its catalytic efficiency, and affect the pH and temperature optima [45].

### 2.3. Thermal Stability of BC-CA-MgFe_2_O_4_ and BC-CA-NiFe_2_O_4_ Carriers by TGA and DSC

**Figure 3:**
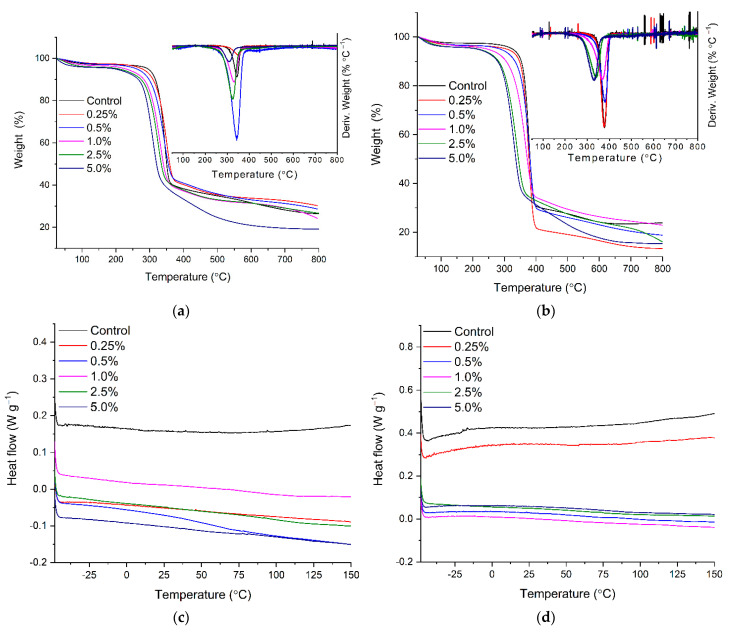
The TGA and DSC analysis results for composites prepared with different concentrations of CA and used ferrites particles NiFe_2_O_4_ (**a**,**c**) and MgFe_2_O_4_ (**b**,**d**).

Thermal properties of the composites were evaluated by thermogravimetric and differential scanning colorimetry and analysis in an inert atmosphere. The obtained results revealed a significant difference in developed materials properties depending on the amount of citric acid used for BC modification and the kind of magnetic particles introduced to its structures (Figure 3a–d). The slight mass loss in all composites at temperature ranges from 30 °C to approximately 180 °C can be assigned to residual water evaporation incorporated in material structure [46]. In this region of TGA spectra for the composites with a higher amount of introduced carboxylic group, slightly higher mass loss was observed. This difference is likely due to the higher affinity of carboxyl moiety to water [41]. The maximum reduction in weight for all composites was visible above 270 °C and can be attributed to BC structure depolymerization due to cleavage of glycosidic linkages between anhydrous glucose molecules. The decomposition temperature was found at the range 335–348 °C (T_onset_), and for non-modified BC coupled with magnetic particles, it was in agreement with the earlier reported value of this parameter for native bacterial cellulose and its composites (Table 1) [47]. The significant shift of decomposition temperature to lower values was observed as closely connected to the increasing amount of CA used for BC modification. The composites obtained through BC modification with citric acid at a concentration above 1.0% exhibited significant shift of T_onset_, respectively, at range 327.2–294.5 °C for BC-CA-MgFe_2_O_4_ and from 295.7 °C to 278.1 °C for BC-CA-NiFe_2_O_4_. The thermostability analysis also showed a significant influence of magnetic particle type on decomposition temperature. Significantly higher decomposition temperatures characterized the BC composites with MgFe_2_O_4_ particles. The thermal stability decrease can be explained by reducing effective hydrogen bonds between BC nanofibers and confirming the changes in its crystallinity after modification by CA [48]. The intended use of new composites as carriers for immobilizing industrially important enzymes requires their stability in the temperature range, usually up to 80 °C [49,50]. Therefore, the results indicate their high stability in the required temperature range (Figure 3c,d) [49].

### 2.4. Magnetic Properties of BC-CA-Ni/Mg Magnetic Carriers

**Figure 4:**
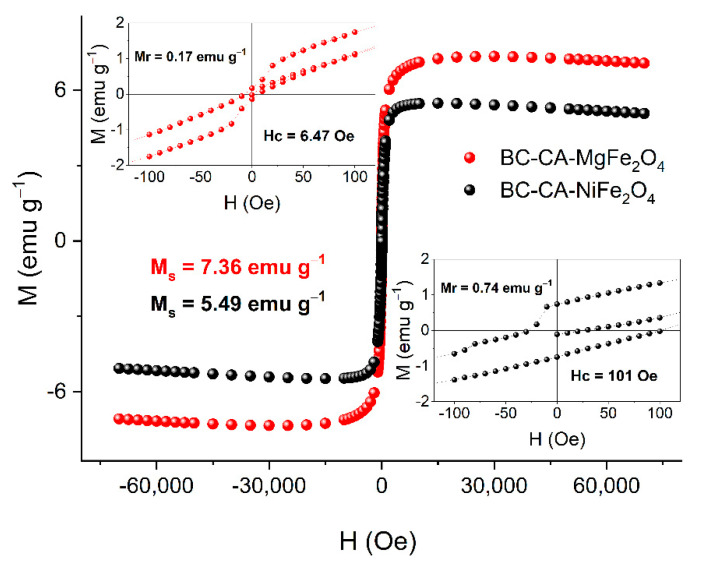
Magnetic hysteresis loop for BC-CA-MgFe_2_O_4_ (●) and BC-CA-NiFe_2_O_4_ (●) of bacterial cellulose magnetic composites.

The hysteresis loop shape indicates the superparamagnetic behavior of the analyzed carrier variant based on nickel and magnesium ferrites. For carrier BC-CA-MgFe_2_O_4_, the saturation magnetization was Ms = 7.36 emu g^−1^ and nearly zero coercivity (Hc = 6.47 Oe), and also zero remanence (Mr = 0.17 emu g^−1^) (Figure 4). Magnesium ferrites exhibit the so-called blocking temperature as a function of magnetic susceptibility and temperature. Above this temperature, superparamagnetic behavior was observed, while below—the ferromagnetic one. At the magnetic field above H = 3 kOe, no blocking temperature was observed for this material. The carrier prepared based on NiFe_2_O_4_ particles exhibited slightly different magnetic properties. For BC-CA-NiFe_2_O_4,_ the saturation magnetization value Ms = 5.49 emu g^−1^ was significantly lower compared to BC-CA-MgFe_2_O_4_. For this variant of the carrier, the magnetic remanence and coercivity values were, respectively, Mr = 0.74 emu g^−1^ and Hc = 101 Oe, suggesting the multidomain structure of nickel ferrites used for BC-CA-NiFe_2_O_4_ preparation. For the second carrier variant, the temperature of magnetic susceptibility was above −223.15 °C and decreased similarly to BC-CA-MgFe_2_O_4_. The magnetic characteristics of developed carriers indicated their different magnetic properties.

### 2.5. Characteristics of the BC-CA Magnetic Composites Morphology

**Figure 5:**
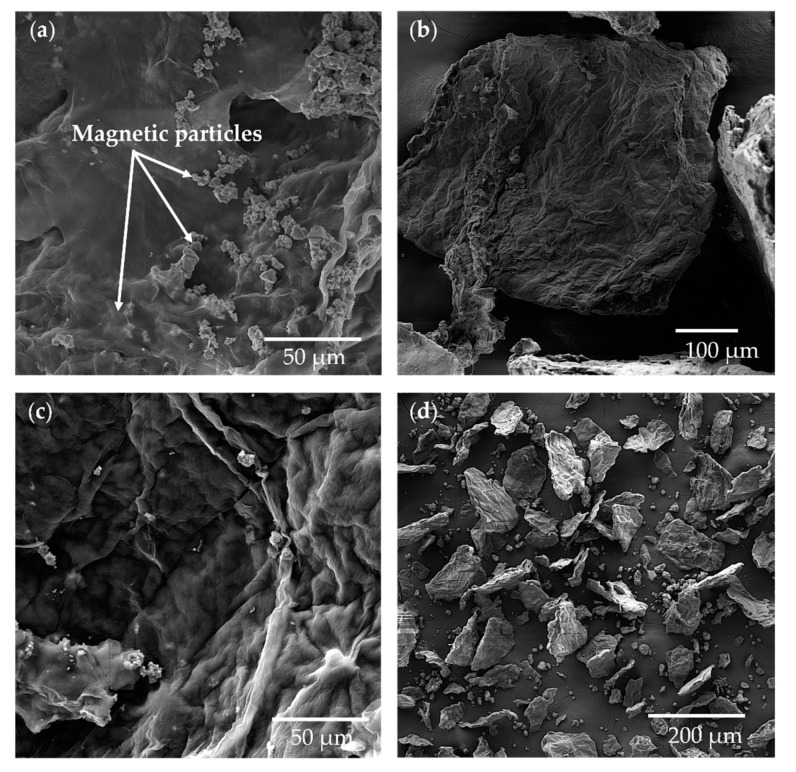
The SEM images of BC modified by 2.5% CA with NiFe_2_O_4_ particles (**a**,**b**) and MgFe_2_O_4_ (**c**,**d**) magnetic particles. The pictures were done with 1000- and 100-times magnification.

Analysis of carriers by scanning electron microscopy was performed to characterize the surface of carriers and visualize their morphology and structural details and assess the influence of modification with citric acid on the BC structure (Figure 5). The carrier particles exhibited different sizes and heterogeneous shapes. The carrier “micro-flakes” surface was decorated with associated magnetic particles, which were also incorporated in deeper parts of the composite structure. The carrier particle’s surface is also slightly undulating, with drawn outlines of agglomerated BC microfibrils. In the unmodified form of BC membranes, the surface is usually characterized by high porosity formed by nanofibers networks (Figure S1). These BC structure properties can influence the enzyme’s immobilization process and final catalytic characteristics [14,51].

### 2.6. Efficiency of Enzymes Immobilization

**Figure 6:**
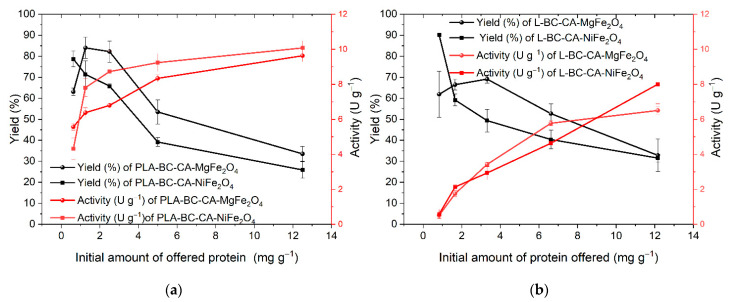
The immobilization yield (%) and activity (units per g of dry carrier mass) of immobilized PLA (**a**) and lipase (**b**) on bacterial cellulose–citric acid-based magnetic carriers.

The efficiency of immobilization and activity of carriers was tested for two enzymes, lipase from *Candida antarctica* and phospholipase A. The immobilization process was performed in the same conditions for both compared enzymes. The immobilization efficiency constantly decreased with the increased amounts of offered protein (Figure 6). The highest activity of PLA was for the highest amount of offered protein. It was 10.07 U g^−1^ and 9.62 U g^−1^, respectively, for nickel- and magnesium-ferrite-based carriers, but with the lowest yield. Similarly, for lipase, the highest activity was detected for the most considerable amount of offered protein (12.0 mg per g of carrier), and it was on average 8.00 U g^−1^ and 6.5 U g^−1^ and had the lowest yield. The best ratio between the final activity of immobilized enzyme and lipase yield was determined at the carrier’s initial protein amount of 6.6 mg g^−1^. The immobilized enzyme exhibited an activity of 5.8 U g^−1^ and 4.6 U g^−1^ for nickel and magnesium-ferrite-based carriers, respectively. Whereas, for PLA, the initial amount of protein 2.5 mg g^−1^ of the carrier was found optimal for nickel-ferrite-based carrier with the activity of 8.7 U g^−1^ and the initial amount of protein 6.6 mg g^−1^ for carrier based on magnesium ferrite with the activity of 8.3 U per g of the carrier. However, the lower amount of offered protein resulted in a higher yield but, as expected, had a lower final activity. This was evident during lipase immobilization.

The distinction in immobilization effectiveness between PLA and lipase is probably a consequence of differences in their structural properties. The PLA is a chimeric enzyme composed in N-terminal part (1–284) from lipase of *Thermomyces lanuginosus* and in C-terminal part (285–339) from phospholipase A1 of *Fusarium oxysporum* [52]. Comparing the amino acid sequence of the lipases from *T. lanuginosus and C. antarctica* showed low similarity and confirmed the high structural divergence of the analyzed enzymes (Figure S2).

### 2.7. Effect of pH on the Activity of Immobilized Enzymes

**Figure 7:**
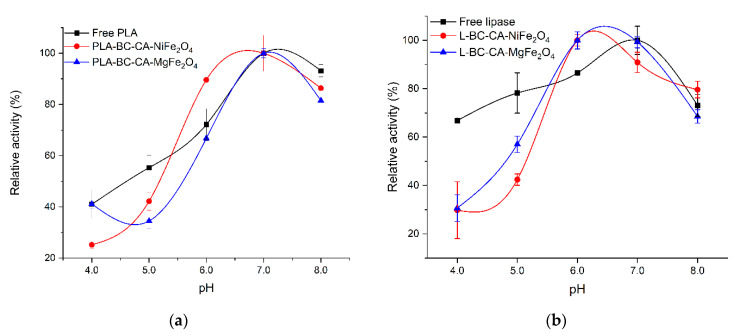
Effect of pH on the activity of free and immobilized carrier variants for phospholipase A1 (**a**) and lipase (**b**).

The immobilization process can also influence on pH activity profile of enzymes. Depending on the carrier structure and, significantly, the charge of functional groups, local pH can differ and manifest in pH optima shift for immobilized enzymes. The immobilization of enzymes on the carriers with a surface abundant in the anionic groups can promote the pH optima shift to alkaline, while for cationic groups to the acidic pH range [53]. After immobilization on both carrier variants, the PLA pH activity profile was not changed significantly with optima at 7.0, similar to a free enzyme (Figure 7a). For lipase immobilized on both carriers, the highest activity was observed at pH range 6.0–7.0, while the free form of the enzyme showed pH optima at pH 7.0.

Moreover, comparing to immobilized lipase, the free enzyme retained more activity at a range below pH 6.0 (Figure 7b). Immobilization of PLA on different carriers, such as cellulose triacetate, caused the pH optima shift to more alkaline [54]. In contrast, PLA immobilization on modified magnetic bacterial cellulose beads did not influence the enzyme pH optima [29]. Immobilization of *Candida rugosa* lipase on a cellulose nanofiber membrane did not change significantly in pH optima either; however, a similar reduction in activity was observed at pH below 6.0 [55]. The observed difference in pH-dependent activity profile between free and immobilized lipase can also result from conformational changes of the enzyme structure. The pH optima of enzymes result from ionizable amino acid pKa values that depend on the active site microenvironment structure [56]. The modification pKa, which is key for the catalytic activity and ionizable amino acid residue in the active center of lipase, probably reduces immobilized enzyme activity at a lower pH than its free form.

### 2.8. Effect of Temperature on Activity and Stability Immobilized Enzyme

**Figure 8:**
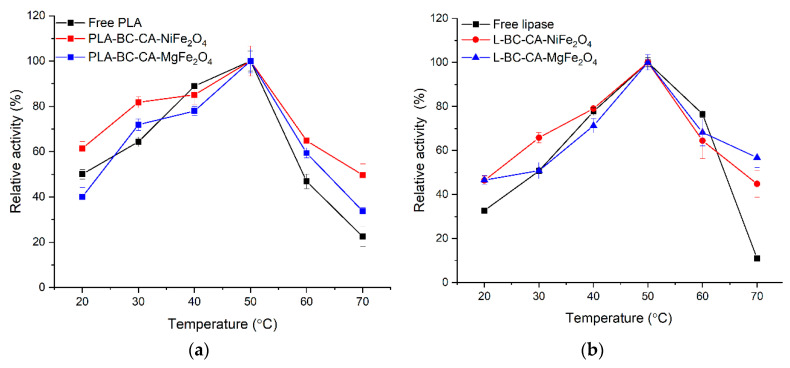
Effect of temperature on the activity of free and immobilized phospholipase A (**a**) and lipase (**b**).

The temperature effect on free and immobilized enzyme activity was analyzed at 20 °C to 70 °C (Figure 8). The phospholipase A and lipase immobilized on both carriers showed a similar free form of enzyme temperature optimum at 50 °C. However, both immobilized enzymes retained a higher level of residual activity at 70 °C, where a significant drop was observed for its soluble form. The thermal stability of both analyzed enzymes was improved (Table 2). At 50 °C, a beneficial effect of immobilization was visible, mainly for lipase. After incubation at 60 °C, free forms of enzymes lost almost 90% of their initial activity, whereas immobilized enzymes on both carriers retained at least 50% of initial activity.

The immobilization process is usually beneficial for enzyme resistance at higher temperatures. For example, immobilization of phospholipase A on cellulose triacetate resulted in improved temperature stability of the enzyme at 50 °C and 60 °C [54]. Drozd et al. [29] did not observe any significant increase in thermal stability of PLA immobilized on polyethyleneimine modified magnetic bacterial cellulose beads. Singh and Mukhopadhyay [57] reported thermal improvement in stability for lipase from *Candida antarctica* immobilized on magnetic cellulose derivates with magnetic properties. Wu et al. [10] for lipase from *Candida rugosa* also found an increase in resistance to high temperatures after immobilization on the carrier based on thermally modified bacterial cellulose. The rational explanation of the thermal stability increasing phenomenon of both immobilized enzymes is reducing their structural flexibility. The hydrogen and covalent bonds formed between the carrier and amino acid residues of enzymes molecule surface can strengthen their rigidity and resistance at higher temperatures [50]. However, a decrease in enzyme structure flexibility can be a reason for reducing their catalytic efficiency, manifested by a reduction in affinity to substrates [58]. Depending on the immobilization protocol and strength of enzyme binding to the carrier, immobilization on the enzyme properties may differ. For this reason, immobilization does not always lead to a positive change in enzyme thermal stability.

### 2.9. Influence of Immobilization Process on Catalytic Constants K_M_ and k_cat_

The immobilization process often influences on K_M_ and k_cat_ constants of enzymes. The K_M_ value of PLA and lipase was not significantly affected by immobilization on the carrier with NiFe_2_O_4_ particles (Table 3). In contrast, for enzymes immobilized on the carrier with MgFe_2_O_4_ particles, where it was almost three times for PLA and two times for lipase, higher K_M_ was observed than for free enzymes. Immobilized PLA on bacterial cellulose beads, modified by Fe_3_O_4_ magnetic particles, also exhibited increased K_M_ [29]. It also indicated the influence of carrier magnetic particles integrated with the structure on the enzyme structure and catalytic efficiency. However, it can also suggest a difference in the structure of carriers. The main reason for changes in catalytic constants is the strength of interaction with carriers that depends on both carrier and enzyme structural properties [59]. Moreover, possible multipoint binding of the enzyme to support often becomes too strong, leading to the rigidification of its structure. Therefore, the enzymes’ substrate specificity is strongly related to their structural flexibility. The limitations in these properties and possible incorrect orientation of enzyme molecule on the support surface can lower the affinity to the substrate, usually manifested by increased K_M_ value [60]. The primary binding site of enzyme molecules in EDC-mediated covalent linkage to BC-CA-NiFe_2_O_4,_ and BC-CA-MgFe_2_O_4_ composites exposed surface carboxyl groups with the amine group of proteins being lysins that are one of the most frequent amino acids on enzymes molecular surface. Moreover, the EDC-mediated coupling of the enzyme to the carrier that is zero-length crosslinkers does not introduce additional atoms of the spacer arm. Finally, the biocatalyst is closely located to the carrier surface [61]. The decreased catalytic efficiency (k_cat_ K_M_^−1^) of phospholipase A and lipase is probably connected with the reducing the functionality of enzyme-specific structural elements [58]. In the case of lipases, their activity strongly depends on the mobility of a characteristic domain containing a secondary structure fragment with α-helix, called a “lid.” The decline in this element enzyme molecule’s ability to significantly move reduces lipase activity by closing access to the active site for the substrate [62].

### 2.10. Reusability

**Figure 9:**
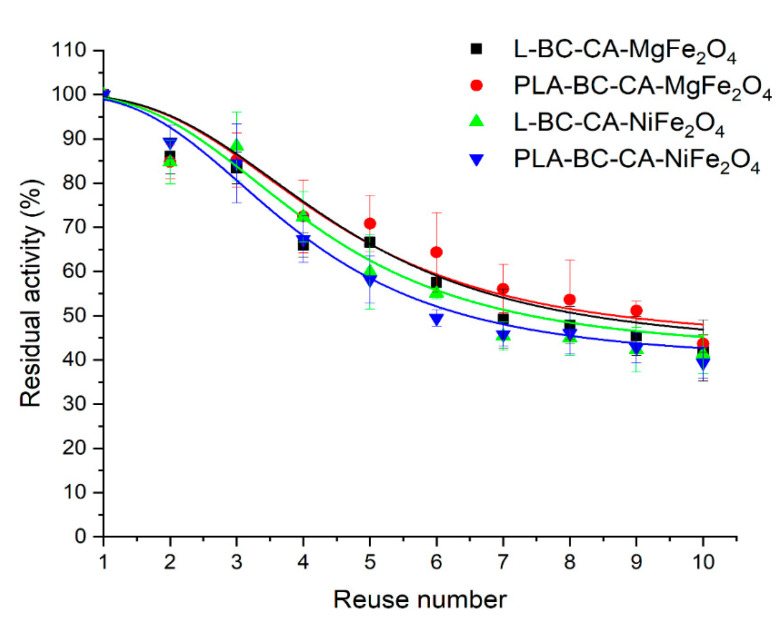
The reusability of PLA and lipase immobilized on different carrier variants.

In practice, the main goal of enzyme immobilization is to reduce costs by the possibility of reusing the immobilized enzymes. The studied enzymes such as lipase and PLA are widely used in industry, and their high stability during repeated uses after immobilization is a desired property [52,63]. The enzymes immobilized on both carrier variants showed good operational stability and, after nine repeated cycles of use, retained more than 50% percent of the initial activity (Figure 9). However, immobilized enzymes’ principal loss of activity was mainly observed after the first cycle of use to almost 75% of initial activity when immobilized on both carrier variants for lipase and between 70% and 60% for PLA. After the first use, a high activity drop was also observed for lipase from *Rhizopus Chinensis* immobilized on modified bacterial cellulose spheres [64]. For the lipase from *Candida rugosa* immobilized on oxidized cellulose nanofiber membrane, a high reduction in activity after the first use was reported as retaining 60% of its initial activity [55]. The porcine pancreatic lipase immobilized on citric acid esterified loofah sponge retained almost 53% of initial activity after nine repeated uses. The highest reduction in activity was observed at initial cycles of use [43]. In earlier studies, PLA immobilized on modified magnetic bacterial cellulose spheres retained almost 70% of initial activity after eight repeated uses. The reduction in activity was also noted after three initial repeated uses [29]. The observed gradual decrease in enzyme activity could be a consequence of the inhibition effect not thoroughly flushing out the residual substrate from the surface of the carrier and the partial inactivation of the enzyme [65].

## 3. Materials and Methods

### 3.1. Chemicals and Reagents

The recombinant lipase *B* from *Candida antarctica* E.C.3.1.1.3 (Lipozyme, CALB-L) and phospholipase A from *Aspergillus oryzae* EC 3.1.1.32 (Lecitase^TM^Ultra) were obtained from Sigma—Aldrich (Poland) and used without further purification. All other chemicals used in the study were at least reagent grade, used without further purification, and ordered from a local supplier of laboratory chemicals.

### 3.2. Bacterial Cellulose Preparation

Prior to the experiment, the 25 mL of 7-day of *Komagataeibacter xylinus* ATCC 53582 culture, was vigorously shaken and then transferred to 2 L of Herstin-Schramm (HS) medium with 20 g L^−1^ glucose as carbon source, 5.0 g L^−1^ yeast extract, 5.0 g L^−1^ bacto-peptone, 1.15 g L^−l^ citric acid, 2.7 g L^−1^ Na_2_HPO_4_, 0.060 g L ^−1^ MgSO_4_•7H_2_O and 1% (*v v*^−1^) of ethanol. The inoculated cultures of *K. xylinus* were maintained in plastic containers (25 cm × 15 cm × 4 cm) filled by 400 mL (2 cm height of medium) of inoculated HS medium for approximately six days at 28 °C. The harvested pellicles were purified from cultivation media and attached cells by three times repeated digestion in 0.1% (*m v*^−1^) NaOH for 30 min at 80 °C. After digestion, the membranes were flushed with deionized water until the flushing’s pH neutralization. The ready membranes were stored at 4 °C until use.

### 3.3. The NiFe_2_O_4_ and MgFe_2_O_4_ Ferrites Particles Preparation

The MgFe_2_O_4_ and NiFe_2_O_4_ magnetic particles were prepared by solid-state reaction at 1000 °C. In the typical synthesis process, the MgCl_2_•6H_2_O or NiCl_2_•6H_2_O was mixed with FeCl_3_•6H_2_O in molar ratio 1:2. After mixing in a ceramic mortar pestle, the formed paste was solidified by heating at 200 °C for 15 min. Then, the sample was transferred to a muffle furnace and sintered at 1000 °C for 3 h. The obtained powder was used for carrier preparation.

### 3.4. The Bacterial Cellulose Citric Acid-Modified Magnetic Composites Preparation

**Figure 10:**
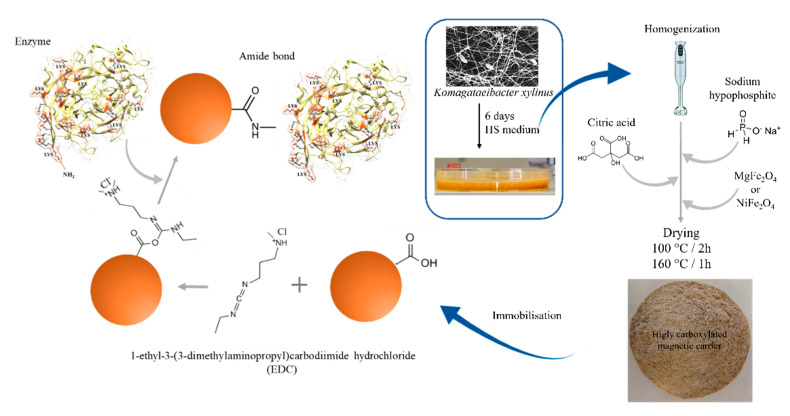
The schema of carrier preparation and enzymes immobilization process.

The bacterial cellulose pellicles after harvesting were homogenized and modified by different concentrations of citric acid in the range from 0.25% to 5.0% (*w w*^−1^) and sodium hypophosphate (SHP) as a catalyst for founding optimal conditions of BC modification (Figure 10). The BC pellicles were cut into small pieces at the first step, blended to obtain homogeneous pulp split to samples with equal mass, and transferred into glass beakers. Next to the pulp was added an appropriate amount of solid CA and SHP in ratio 1:0.5 and magnetic material 0.5% (*w w*^−1^). Next, the mixture was again blended to complete dissolution and dispersion of additives. The obtained mixture was spread on the Teflon mats with ≈2–3 mm thickness and transferred to a laboratory oven for 2 h at 100 °C until water evaporation. Then, the temperature was increased to 160 °C for 1 h. The dried material was powdered in a scissor laboratory grinder and finally flushed in deionized water until pH stabilization and dried to constants mass in a laboratory oven at 60 °C.

### 3.5. Carriers Properties Determination

#### 3.5.1. The Carboxyl Group Content Assay at Carrier Surface

The carboxyl content assay was performed according to a modified protocol [66]. The wet sample of the carrier was incubated with 1 mL of 0.1% (*w w*^−1^) solution of toluidine blue (TB) in 1 mmol L^−1^ NaOH for 15 min at 40 °C with periodical vigorous shaking. Next, carrier samples were collected from TB solution using a magnetic separator, and the supernatant was discarded. The separated carrier samples were rinsed at least two times by 1 mL of 1 mmol L-1 NaOH solution to remove excess of TB until the time of colorless the flushing’s appearing. Next, TB was desorbed by filling the carrier sample in 1 mL of 20% (*w w*^−1^) of sodium dodecyl sulfate solution in deionized water, for 30 min at 40 °C, with periodical vigorous shaking. Next, 300 µL of supernatant was transferred to a microplate cell, and absorbance was measured by Tecan Infinite 200 Pro microplate reader at 625 nm. The amount of carboxylic group on the surface of the carrier was expressed as mmol of COOH per gram of dry mass of the carrier.

#### 3.5.2. Scanning Electron Microscopy (SEM)

The SEM analysis of carriers’ surface was performed using a high-resolution field emission gun scanning electron microscope VEGA3 TESCAN. Prior to the analysis, samples of the carrier were dried and then fixed by the sputtering with Au/Pd (60:40) using the Q150R ES device.

#### 3.5.3. Attenuated Total Reflectance Fourier Transform Infrared Spectral Studies (ATR-FTIR)

Before ATR-FTIR analysis, samples of not modified bacterial cellulose and composites were dried in an Eppendorf tube at 60 °C. Next, the analysis was performed using a Bruker spectrophotometer with an ATR-FTIR adapter. The spectra were collected in the range of 4000–400 cm^−1^ with a resolution of 8 cm^−1^ and 64 scans. The obtained ATR-FTIR spectra were analyzed using the SpectraGryph 1.2 and 2D SHIGE software for generation 2D correlation matrix of spectra and visualized in Origin2021pro.

#### 3.5.4. Differential Scanning Calorimeter Analysis (DSC)

Determination of the thermal transitions of the carrier was performed in a Mettler Toledo DSC 1 STARe differential scanning calorimeter, coupled with a Huber TC100 immersion cooler. The calorimeter was calibrated for temperature and cell constants using indium. All data were collected at atmospheric pressure, with nitrogen as a purge gas and an empty sample pan as the reference. Samples used were between 6 and 15 mg. In the first heating cycle, the heating ramp was set from −50 °C to 400 °C with a heating rate of 10 °C min^−1^. At this temperature, samples were held for 5 min isotherm. In the next step, samples were cooled from 125 °C to −80 °C with a cooling rate of 10 °C min^−1^ and then were kept for 10 min at −80 °C. The last step was heating the sample from −80 °C back to 125 °C with a heating rate of 10 °C min^−1^. The data from the DSC thermograms were obtained via analysis with the STARe Evaluation Software by Mettler Toledo.

#### 3.5.5. Thermogravimetric Analysis (TGA)

The thermal stabilities of the carrier were analyzed by using TGA Q50 thermogravimetric analyzer. The TGA experiment was conducted under a nitrogen atmosphere and measured in the dynamic heating regime. Samples between 5 and 10 mg were heated from 25 °C to 500 °C with a heating rate of 3.5 °C min^−1^ with a 10 min. isotherm at 85 °C under nitrogen atmosphere to remove any remaining water present in the samples. Decomposition temperatures of the composite were established as the onset temperature for decomposition of the first 5% of the sample (T5%onset) and as the regular onset temperature for decomposition (Tonset) for the whole sample or each of the consecutive steps in multistep decompositions.

#### 3.5.6. Static (Dc) Magnetic Susceptibility Measurements of BC-CA-Ni/Mg Ferrite Composites

Static (Dc) magnetic susceptibility measurements were carried out using Quantum Design MPMS XL-7 with superconducting quantum interference device (SQUID) magnetometer at temperatures from 2 K to 300 K and magnetic field of 3000 Oe. A magnetic hysteresis loop was performed up to 70 kOe. Dc susceptibility measurements were performed in zero-field cooling (ZFC) mode.

### 3.6. Enzymes Immobilization

#### 3.6.1. Lipase and Phospholipase a Assay of Hydrolytic Activity

The 4-nitrophenol butyrate (*p*NPB) was used as the substrate to determine lipase and phospholipase A hydrolytic activity, at a concentration of 0.5 mmol L^−1^ in 50 mmol L^−1^ phosphate buffer pH 7.0 with 0.004% (*v v*^−1^) Triton X-100. The working substrate solution was prepared from 5 mmol L^−1^ stock solution of *p*NPB in 2-propanol, diluted in 10 volumes of working buffer, shortly before activity measure to minimize background of the assay. The activity was determined by measuring the absorbance changes at wavelength λ = 348 nm (ε = 5.400 L mmol^−1^ cm^−1^) using a microplate reader TECAN Infinite 200 Pro for 5 min at 30 °C. One phospholipase A or lipase unit releases 1 µmol *p*NP per minute at 30 °C and pH 7.0. The specific activity of the free enzymes was expressed as units per mg protein and immobilized enzyme as units per g of dry carrier mass.

#### 3.6.2. Enzymes Immobilization

Before enzymes immobilization, the carrier was transferred to Eppendorf tubes and then filled in 25 mmol L^−1^ MES (2-(N-morpholino)ethanesulfonic acid) buffer pH 5.0 for 30 min. Next equilibrated carrier was collected, and the buffer was replaced by a fresh solution of 1-ethyl-3-(3-dimethylaminopropyl)carbodiimide (EDC) 50 mg mL^−1^ in the same buffer and left on a roller shaker for 1 h. After activation time, the carrier was again collected, quickly flushed with the buffer. Finally, 1 mL of enzyme solution in MES buffer was transferred to a tube and incubated for 2 h with mixing on a roller shaker. Next, the carrier with immobilized enzymes was collected using a magnetic separator and flushed with 1 mL of MES buffer and 50 mmol L^−1^ sodium phosphate buffer pH 7.0. The efficiency of immobilization was determined with enzymes solution in the protein concentration range from 0.25 mg mL^−1^ to 0.005 mg mL^−1^ per 20 mg of dry carrier mass. The immobilization yield (%) was calculated according to Equation (1).

(1) $$\mathrm{Immobilization}\;\mathrm{yield}\;\left(\%\right)=\left(\frac{\left(A_{i}-A_{R}\right)}{A_{i}}\right)\times 100$$

where *A_i_* is an initial amount of enzymes activity units offered to immobilization, *A_R_* is a sum of residual enzymes’ activity units from the supernatant and subsequent steps of immobilization protocol [67].

#### 3.6.3. Protein Concentration Determination

Protein concentration of enzymes preparation was assayed by the Bradford method with bovine serum albumin as a standard [68].

#### 3.6.4. Effect of Temperature and pH on Free and Immobilized Enzymes

The pH optimum for free and immobilized enzymes, the enzyme activity was determined at pH range 4.0 to 8.0 with 50 mmol L^−1^ acetate buffer pH 4.0–5.0, 50 mmol L^−1^ phosphate buffer pH 6.0 to 8.0. The temperature optimum was measured at 20 °C, 30 °C, 40 °C, 50 °C, 60 °C, and 70 °C. Before adding the enzyme, the substrate solution was equilibrated to the appropriate temperature, and next, 10 μL of the enzyme solution was added to 300 μL of the substrate and incubated for 5 min. Determination of the optimum temperature for the immobilized enzymes was carried out by transferring 500 μL preheated substrate solution to the tube with 10 mg of the immobilized enzyme. The mixture was then incubated for 5 min at appropriate temperatures. After separating immobilized enzymes, the 300 μL of the reaction solution was transferred to a cell on a microplate for absorbance recording. The assay of thermal stability of free enzymes was done by incubation of its solution in 50 mmol L^−1^ phosphate buffer pH 7.0 at temperature 50 °C and 60 °C for 30 min. The thermal stability of immobilized enzymes was determined by incubating 10 mg of immobilized enzyme in 500 µL of 50 mmol L^−1^ phosphate buffer, pH 7.0. After incubation, the activity of preparation was determined by the standard protocol at reference temperature 30 °C. The residual activity was expressed in relative terms with free and immobilized enzymes activity at a reference temperature of 100%.

#### 3.6.5. Determination of Kinetic Parameters of Immobilized Enzymes

The kinetic parameters for both free and immobilized enzymes were determined by measuring the reaction rates at different concentrations of substrate *p*NPB at range 0.1 to 1.2 mmol L^−1^ at 30 °C and pH 7.0. The Michaelis-Menten constant (K_M_) and maximal reaction rate V_max_ were calculated according to the Michaelis-Menten equation via a general nonlinear least square fitting using OriginPro 2021 software. (Figure S3).

#### 3.6.6. Reusability of Immobilized Enzymes

The operational parameter as reusability of the immobilized enzymes was carried out in optimal conditions as for activity assay for each analyzed enzyme at 30 °C and was repeated ten times. After every cycle, the immobilized enzymes were recovered from the reaction mixture using a magnetic separator and flushed twice with 50 mmol L^−1^ phosphate buffer pH 7.0. Next, recovered immobilized enzymes were reused again with the use of a fresh buffered-substrate solution. The initial activity of the immobilized enzymes was defined as 100%.

## 4. Conclusions

The new magnetic carriers for enzyme immobilization by efficient carboxylation of bacterial cellulose using citrate were developed. The carriers were characterized by a high density of the carboxylic groups, significant thermal stability, and magnetic suspensibility depended on the kind of used magnetic particles. The degree of modification of bacterial cellulose by CA, allowed enzymes to be immobilized efficiently using the simple protocols which apply only EDC as the activation regent. The immobilized enzymes exhibited an improved operational stability at higher temperatures and reduced their catalytic efficiency compared to enzyme free forms compensated by relatively high resistance for repeated uses. Moreover, the introduction to the carrier structure of different types of magnetic particles allows for easy separation of immobilized enzymes from the reaction vessel. Apart from the mentioned advantage of newly developed carriers, they can be used as efficient support when immobilized enzymes are used in magnetic field-supported reactors. Nowadays, these kinds of reactors are gaining more and more importance in many industries where direct controlling of the biocatalyst action is strongly required. The developed carriers also possess a high potential for further modification with various available spacers arms for designing other magnetic carriers with desired properties to immobilize other enzymes with high industrial importance.

## Figures and Tables

**Table 1:** Thermal stability (TGA) of prepared BC-CA-based magnetic composites.

Initial CA Concentration (%)	BC-CA- NiFe_2_O_4_			BC-CA- MgFe_2_O_4_		
	T_onset_	T_max_	* Mass Loss (%)	T_onset_	T_max_	* Mass Loss (%)
	(°C)			(°C)		
0	335.1	355.5	59.6	348.7	392.8	66.7
0.25	317.9	365.1	61.1	356.2	390.7	68.4
0.5	305.8	360.7	60.1	351.2	393.7	70.9
1.0	295.7	349.1	59.9	327.2	386.1	66.4
2.5	289.0	340.5	59.8	297.0	358.6	66.5
5.0	278.1	327.7	60.3	294.5	349.4	66.2

* The mass loss was calculated according to mass value at the temperature of 500 °C for BC-CA- NiFe_2_O_4_ and 800 °C for BC-CA-MgFe_2_O_4_.

**Table 2:** Effect of temperature on the stability of free and immobilized enzymes.

	Temperature	
	50 °C	60 °C
	Relative Activity (%)	
Free PLA *	82.0 ± 4.0	12.4 ± 0.2
Free Lipase	45.2 ± 2.1	18.9 ± 1.6
PLA-BC-CA-NiFe_2_O_4_	89.4 ± 3.7	56.5 ± 7.6
PLA-BC-CA-MgFe_2_O_4_	77.4 ± 6.7	63.1 ± 2.2
L-BC-CA-NiFe_2_O_4_	60.4 ± 3.2	50.5 ± 12.6
L-BC-CA-MgFe_2_O_4_	87.4 ± 4.6	66.5 ± 2.1

* Free and immobilized enzymes were incubated in phosphate-buffered solution for 30 min, and residual activity was measured at 30 °C according to standard protocol.

**Table 3:** The values of catalytic constants of immobilized enzymes on both carrier variants.

	^a^ K_M_ (mmol L^−1^)	k_cat_ (s^−1^)	k_cat_ K_M_^−1^ (L mmol^−1^ s^−1^)
Free PLA	0.49 ± 0.10	57.6 ± 2.5 × 10^7^	119.94 ± 22.34 × 10^7^
Free lipase	0.16 ± 0.01	3.07 ± 0.24 × 10^7^	19.56 ± 0.43 × 10^7^
PLA-BC-CA-NiFe_2_O_4_	0.43 ± 0.09	39.78 ± 3.74	92.40 ± 3.7
PLA-BC-CA-MgFe_2_O_4_	1.49 ± 0.08	44.68 ± 1.56	39.78 ± 9.5
L-BC-CA-NiFe_2_O_4_	0.19 ± 0.03	39.78 ± 3.74	147.05 ± 17.36
L-BC-CA-MgFe_2_O_4_	0.29 ± 0.03	28.13 ± 1.21	30.07 ± 1.91

^a^ for substrate *p*-nitrophenyl-butyrate.

## Data Availability

The data presented in this study are available on request from the corresponding author.

## Supplementary Materials

The following are available online at https://www.mdpi.com/article/10.3390/ijms22168563/s1.
