# Supplementary material for: The Simple Method of Preparation of Highly Carboxylated Bacterial Cellulose with Ni- and Mg-Ferrite-Based Versatile Magnetic Carrier for Enzyme Immobilization
Source: Int J Mol Sci. 2021 Aug 9;22(16):8563. doi: 10.3390/ijms22168563 (PMC8395317; doi:10.3390/ijms22168563)
Supplement: Supplementary file 1 [file ijms-22-08563-s001.zip › Figure_S1.pdf]

The Simple Method of Preparation of Highly Carboxylated Bacterial Cellulose with Ni- and Mg-Ferrite-Based Versatile Magnetic Carrier for Enzyme Immobilization

Radosław Drozd, Magdalena Szymańska, Katarzyna Przygrodzka, Jakub Hoppe, Grzegorz Leniec, Urszula Kowalska

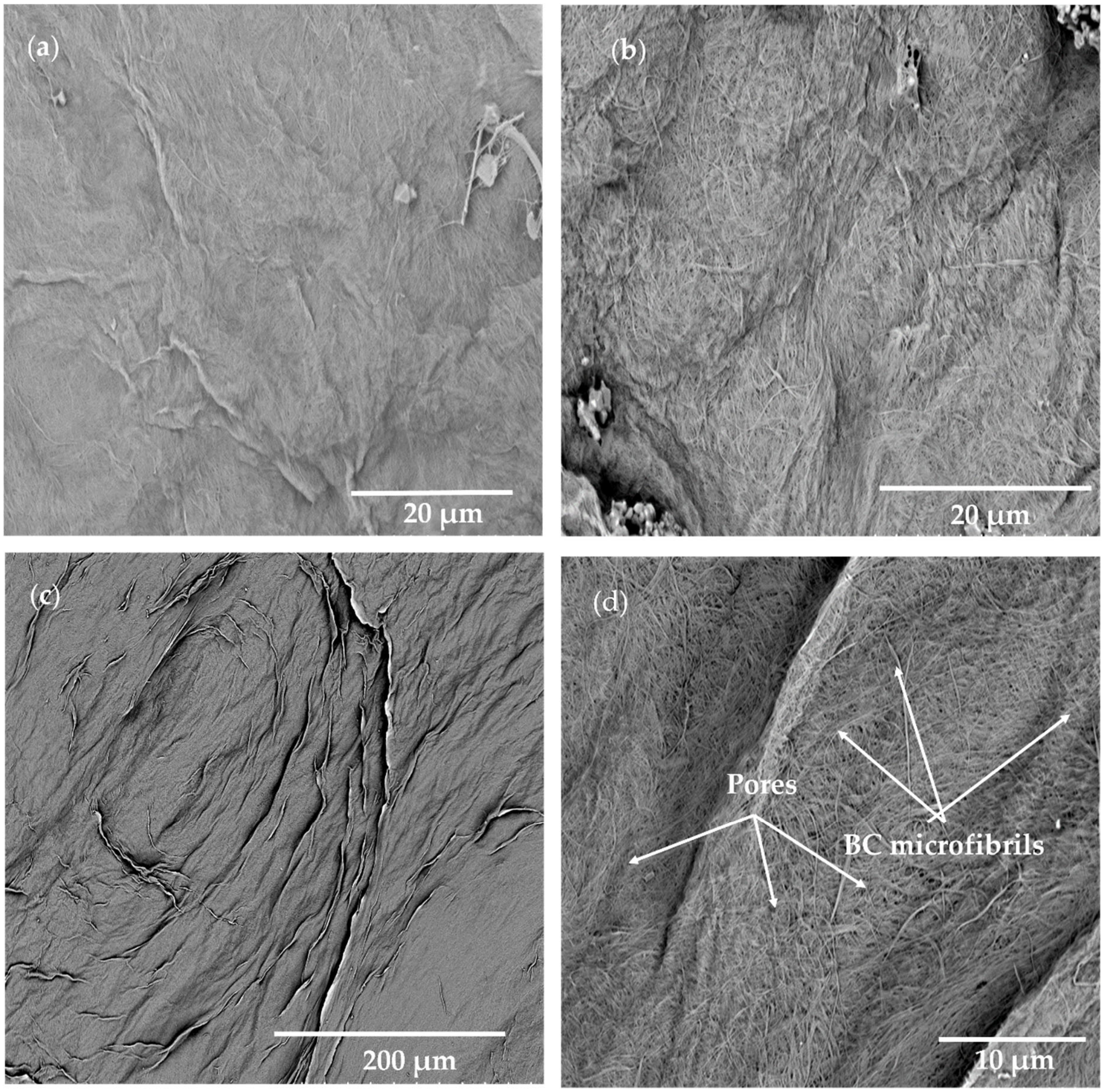

Figure S1. The SEM image of surface BC-CA-$NiFe_2O_4$ (**a**), surface of BC-CA-$MgFe_2O_4$ (**b**), surface not modified BC pulp (**c,d**).
