# Supplementary material for: The Simple Method of Preparation of Highly Carboxylated Bacterial Cellulose with Ni- and Mg-Ferrite-Based Versatile Magnetic Carrier for Enzyme Immobilization
Source: Int J Mol Sci. 2021 Aug 9;22(16):8563. doi: 10.3390/ijms22168563 (PMC8395317; doi:10.3390/ijms22168563)
Supplement: Supplementary file 1 [file ijms-22-08563-s001.zip › Figure_S2.pdf]

The Simple Method of Preparation of Highly Carboxylated Bacterial Cellulose with Ni- and Mg-Ferrite-Based Versatile Magnetic Carrier for Enzyme Immobilization

Radosław Drozd, Magdalena Szymańska, Katarzyna Przygrodzka, Jakub Hoppe, Grzegorz Leniec, Urszula Kowalska

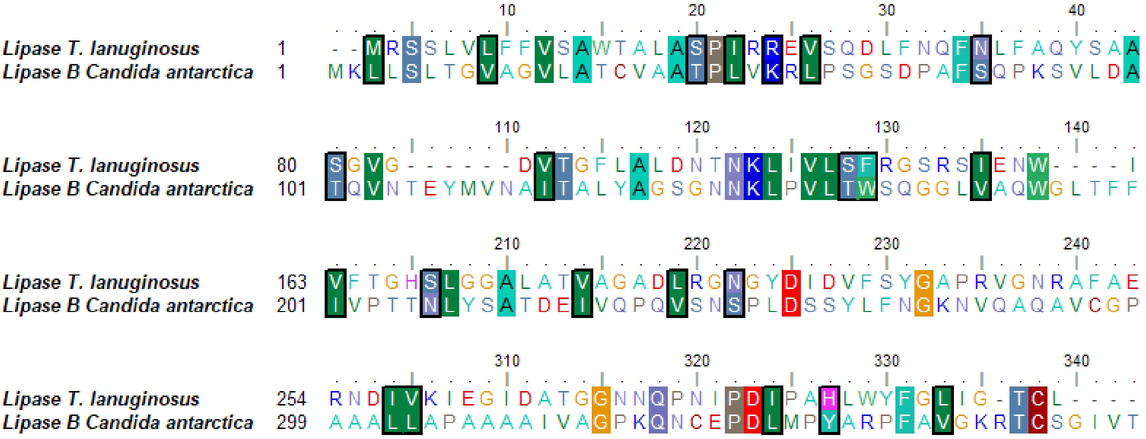

Figure S2. The pairwise comparison of amino acid sequences of lipase from *Thermomyces langinousus* (SwissProt; O59952) and lipase B from *Candida antarctica* (SwissProt; P41365). The alignment was done with using ClustalW via BioEdit software. The compared aminoacid sequences of lipases shares a low level of similarity with a few short consensus region (shadowed letters).
