# Supplementary material for: The Simple Method of Preparation of Highly Carboxylated Bacterial Cellulose with Ni- and Mg-Ferrite-Based Versatile Magnetic Carrier for Enzyme Immobilization
Source: Int J Mol Sci. 2021 Aug 9;22(16):8563. doi: 10.3390/ijms22168563 (PMC8395317; doi:10.3390/ijms22168563)
Supplement: Supplementary file 1 [file ijms-22-08563-s001.zip › Figure_S3.pdf]

# The Simple Method of Preparation of Highly Carboxylated Bacterial Cellulose with Ni- and Mg-Ferrite-Based Versatile Magnetic Carrier for Enzyme Immobilization

Radosław Drozd, Magdalena Szymańska, Katarzyna Przygrodzka, Jakub Hoppe, Grzegorz Leniec, Urszula Kowalska

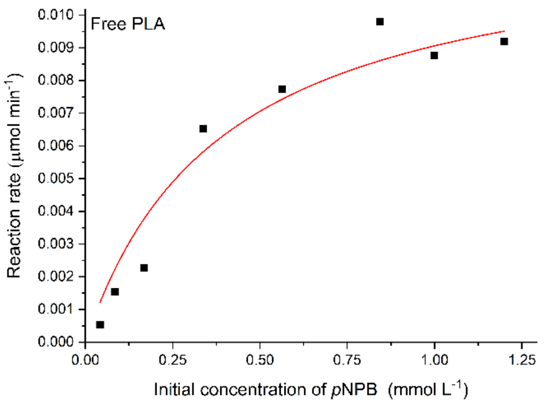


(**a**)

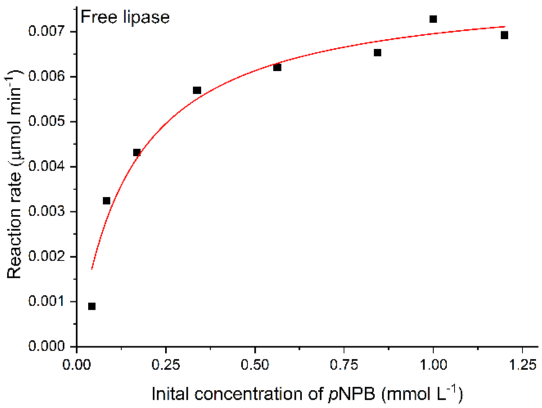


(**b**)

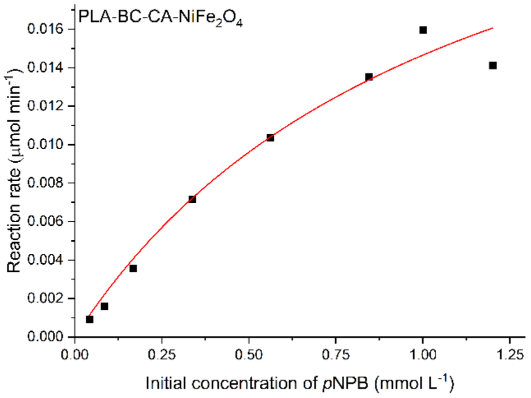


(**c**)

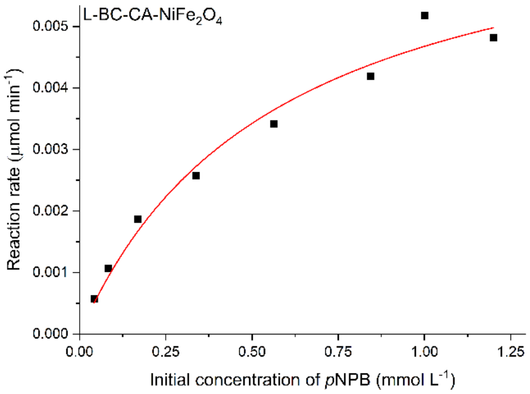


(**d**)

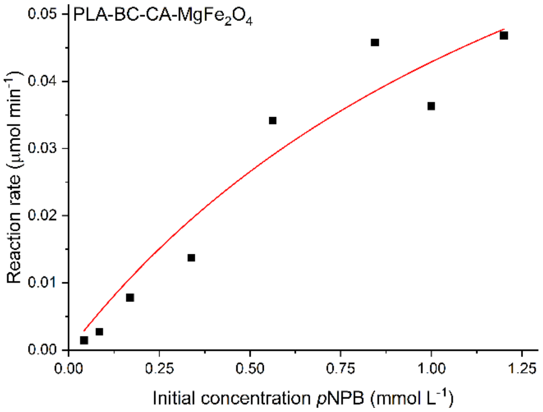


(**e**)

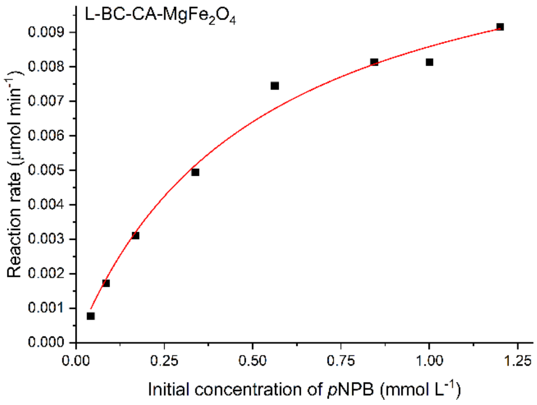


(**f**)

**Figure S3**. The representative dependences of reaction rate vs initial substrate concentration used for determination of catalytic constants of free and immobilized enzymes.
